# A FDR Sensor for Measuring Complex Soil Dielectric Permittivity in the 10–500 MHz Frequency Range

**DOI:** 10.3390/s100403314

**Published:** 2010-04-05

**Authors:** Wojciech Skierucha, Andrzej Wilczek

**Affiliations:** Institute of Agrophysics, Polish Academy of Sciences, Doświadczalna 4, 20-290 Lublin, Poland; E-Mail: a.wilczek@ipan.lublin.pl

**Keywords:** FDR, TDR, dielectric permittivity, dielectric spectroscopy, soil moisture, soil salinity

## Abstract

Mechanical details as well as electrical models of FDR (frequency domain reflectometry) sensors for the measurement of the complex dielectric permittivity of porous materials are presented. The sensors are formed from two stainless steel parallel waveguides of various lengths. Using the data from VNA (vector network analyzer) with the connected FDR sensor and selected models of the applied sensor it was possible obtain the frequency spectrum of dielectric permittivity from 10 to 500 MHz of reference liquids and soil samples of various moisture and salinity. The performance of the analyzed sensors were compared with TDR (time domain reflectometry) ones of similar mechanical construction.

## 1. Introduction

The requirements for fast, reliable and automated spatially distributed soil water content monitoring are not fully satisfied by the current commercially available technology measurements. The reasons for that are high price of commercially available TDR meters working in the frequency range up to 1 GHz, the need for site specific calibrations of FDR meters, especially for heavy and saline soils, or problems with disposal of radioactive elements when the neutron scattering method is used. Scientists and engineers are conducting research and building prototypes to develop new soil water and salinity sensors and their physical models to increase measurement selectivity, accuracy, ease of operation and availability, as well as to make them economically accessible for mass applications. Nondestructive and *in situ* measurements of soil water content and salinity are fundamental in many agricultural applications including hydrology, precision agriculture and irrigation scheduling [1–3]. The idea of applying FDR sensors working in the frequency range above 150 MHz, which is the upper frequency limit of currently available sensors, and reaching 500 MHz, seems to be promising, especially to achieve more universal calibration than current FDR sensors and lower cost than the current TDR meters [4].

At present, several methods are considered suitable for continuous soil water monitoring at the point scale with minimum soil disturbance. Such methods are based on the dielectric properties of soil and the propagation characteristics of electromagnetic (EM) waves in the soil, which change with water content and salinity. A review of soil moisture and electrical conductivity measurement methods, including the dielectric methods at different spatial and temporal scales is presented in [4]. The relationship between the real part of the permittivity and soil volumetric water content is strong for the quartz-dominated soils [5]. Although quartz-dominated materials show negligible frequency dependence of dielectric properties, some clay minerals present dielectric dispersion [6–8]. As a result, the relationship between soil moisture and electrical conductivity depends on the operating frequency of the applied sensor. The least frequency dependence occurs above 500 MHz [7]. Also, accurate measurement of the real part of the soil complex dielectric permittivity is required to fully characterize its relationship with soil moisture in the frequency domain.

The aim of the study is to analyze the influence of soil water content and electrical conductivity/salinity on the on the complex dielectric permittivity of soil measured by a developed FDR (Frequency Domain Reflectometry) sensor and the measurement setup. The sensor and its physical models are used to measure the complex dielectric permittivity in the 10–500 MHz frequency range. The measurements are made in laboratory conditions using specialized equipment not suitable for field use. The presented study is an initial step in works on field sensors and a prototype of a field meter for the measurement of dielectric permittivity of soil in this frequency spectrum and hopefully up to 1 GHz.

## 2. Sensor Description and Measurement Tools

**Figure 1.**
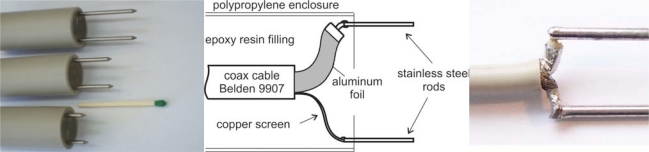
Two wire stainless steel rod FDR probes (left), the scheme of internal details (middle) and the interface between coaxial and parallel waveguides (right).

The applied dielectric sensors were built with two stainless steel rods of 2 mm in diameter, 13 mm separation distance and lengths of 1, 2 and 3 cm (Figure 1—left). The interface between the coax cable and stainless steel rods was constructed to minimize the impedance discontinuity (Figure 1—right).

The mechanical construction of these sensors is similar to the FP/m type manufactured in IA PAS (Lublin, Poland) working with the TDR technique [9]. TDR probes with such short length are not used because of timing restrictions imposed by this measurement method. The presented FDR probes with parallel 1–3 cm length rods may fill the gap for dielectric sensors between open-ended coax [10] and TDR sensors. The TDR measurements give averaged information about the apparent dielectric permittivity and electrical conductivity of soil for a broad range of frequencies dependent n the rise time of the analyzing pulse. In order to receive detailed frequency relations of the complex dielectric permittivity it is necessary to apply FDR technique with appropriate models of the measurement sensors. Several dielectric models of the constructed FDR probe were tested to select the one, which performance on the reference liquids with known dielectric properties was superior over the others.

### 2.1. The Sensor Rods as a Loss Capacitor—the C_0_ Capacitance Model

This model is based on the open-ended coax sensor [11]. This type of sensor is very popular because of the availability of an Agilent 85070E open-ended coaxial probe [12], which connected to an Agilent Vector Network Analyzers equipped with specialized firmware, can produce a frequency spectrum of the real and imaginary parts of the dielectric permittivity of the medium that is in contact with this probe. Open-ended coax probes can be inserted in it, or used for non-invasive surface measurements.

**Figure 2.**
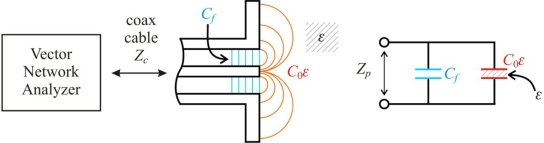
Capacitance model *C_f_* – *C*_0_ of an open-ended coax sensor.

The termination of the open-ended sensor is described by capacitances *C_f_* and *C*_0_*ε*, where the former is the fringing capacitance inside the coaxial waveguide of characteristic impedance *Z_C_* = 50 Ω and the latter is the fringing capacitance due to the test sample (Figure 2). The value of *C*_0_ represents the case when the surroundings of the *C*_0_*ε* capacitor is air.

The values of capacitance and loss of the *C*_0_*ε* capacitor change with the real Re(*ε*) and imaginary Im(*ε*) parts of the complex dielectric permittivity of the tested sample. The values of *C_f_* and *C*_0_*ε* are determined for each angular frequency *ω* = 2*πf* during the calibration process using dielectric media of tabularized dielectric properties. The sensor impedance *Z_p_* is:

(1) $$Z_{p}=\frac{1}{j\omega \left(C_{f}+C_{0}\varepsilon \right)}$$

The Vector Network Analyzer (VNA) measures the reflection coefficient *S*_11_ defined as:

(2) $$S_{11}=\left|S_{11}\right|\;e^{j\varphi }=\frac{Z_{p}-Z_{C}}{Z_{p}+Z_{C}}$$

where |*S*_11_| and φ are the module and phase of the complex reflection coefficient *S*_11_ measured by the VNA.

Real and imaginary parts of *ε* can be found after inserting (1) into (2) as shown below [13]:

(3) $$\text{Re}(\varepsilon )=\frac{-2\left|S_{11}\right|\text{sin}\varphi }{\omega C_{0}Z_{C}\left(1+2\left|S_{11}\right|\text{cos}\varphi +{\left|S_{11}\right|}^{2}\right)}-\frac{C_{f}}{C_{0}};\;\text{Im}(\varepsilon )=\frac{1-{\left|S_{11}\right|}^{2}}{\omega C_{0}Z_{C}\left(1+2\left|S_{11}\right|\text{cos}\varphi +{\left|S_{11}\right|}^{2}\right)}$$

**Figure 3.**
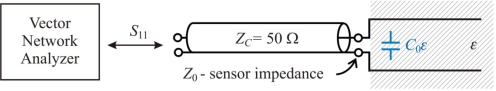
Capacitance model *C*_0_ of a two-wire sensor in lossy dielectric medium.

The capacitance *C*_0_ in a two wire sensor is much bigger as compared to *C_f_*, which is not true for an open-ended coax probe and *C_f_* can be neglected [11] as presented in Figure 3.

The impedance of the *C*_0_ capacitor filled with lossy dielectric of the complex dielectric permittivity equal to *ε* is:

(4) $$Z_{0}=\frac{1}{j\omega C_{0}\varepsilon }=\frac{Z_{\mathrm{air}}}{\varepsilon }$$

where *Z_air_* is the impedance of an air capacitor. The impedance *Z*_0_ of a two-wire sensor at the end of a coaxial cable of characteristic impedance *Z_C_* is given as:

(5) $$Z_{0}=Z_{C}\frac{1+S_{11}}{1-S_{11}}$$

The formula for calculating the complex dielectric permittivity *ε* of the measured medium can be found after inserting (4) into (5):

(6) $$\varepsilon =\frac{\left(1+S_{11\mathrm{air}}\right)\left(1-S_{11m}\right)}{\left(1-S_{11\mathrm{air}}\right)\left(1+S_{11m}\right)}$$

where *S*_11*air*_ and *S*_11*m*_ are complex reflection coefficients measured by VNA in air and the measured medium.

The capacitance model *C*_0_ does not include the effect of electromagnetic radiation from the sensor open ending, which increases with the applied frequency *f* of the measurement signal. The radiation increases the loss of the capacitor *C*_0_ and in consequence increases the imaginary part Im(*ε*) of the tested dielectric permittivity.

### 2.2. Four-Pole T Model of the Open-Ended Coax Sensor

**Figure 4.**
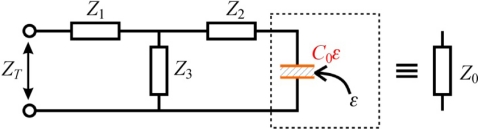
Four-pole T model of the open-ended coax sensor.

A four-pole T model of the open ended coax sensor was described in [14] and it is a modified capacitance model *C*_0_, where the capacitance *C_f_* is replaced by a four pole type T in the form of three impedances *Z*_1_, *Z*_2_, *Z*_3_ (Figure 4).

The impedance of the whole set can be substituted by *Z_T_* and rewritten in the linear form with complex coefficients *a* = *Z*_1_*Z*_2_ + *Z*_2_*Z*_3_ + *Z*_3_*Z*_1_, *b* = *Z*_1_ + *Z*_2_ and *c* = *Z*_2_ + *Z*_3_ and then rewritten again to incorporate the impedance of the sensor in air *Z_air_*:

(7) $$Z_{T}=Z_{1}+\frac{Z_{3}\left(Z_{2}+Z_{0}\right)}{Z_{2}+Z_{3}+Z_{0}}\Leftrightarrow a+{\mathrm{bZ}}_{0}-{\mathrm{cZ}}_{T}=Z_{T}\;Z_{0}\Leftrightarrow \frac{a}{Z_{\mathrm{air}}}+\frac{b}{\varepsilon }+\frac{{\mathrm{cZ}}_{T}}{Z_{\mathrm{air}}}=\frac{Z_{T}}{\varepsilon }$$

*Z_T_* can be calculated from the values of *S*_11_ measured by the VNA using Equation (5), where *Z_T_* will replace Z_0_.

The parameters *a*, *b* and *c* can be determined by the measurement of *Z_T_* in three defined conditions:

– short circuit of the sensor by means of mercury when Re(*ε*) = ∞ and Im(*ε*) = ∞,
– measurement in air when Re(*ε*) = 1, Im(*ε*) = 0,
– measurement in a liquid that has well defined dielectric properties by Deby’e or Cole-Cole models, ex. distilled water, acetone or methanol (Table 2).

Having the parameters *a*, *b*, *c* from above described calibrations it is possible to calculate the complex value of *ε* after rearranging the Equation (7).

### 2.3. Impedance Model Z_f_ – Z_0_

**Figure 5.**
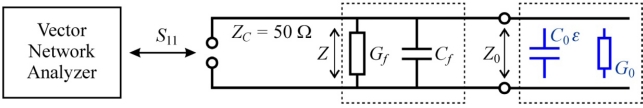
Localization of the sensor impedances in the model *Z_f_* – *Z*_0_.

In this model the capacitances *C_f_* and *C*_0_ (Figure 2) are replaced with the impedances *Z_f_* and *Z*_0_ because they include the conductances *G_f_* and *G*_0_ describing the sensor attenuation and the loss of the measured medium, respectively (Figure 5).

The impedance values of *Z_f_* and *Z*_0_ are determined for each measurement frequency during the calibration process, similarly to the *C_f_* and C_0_ values of an open-ended coax probe. The substitute impedance *Z* of the impedances *Z_f_* and *Z*_0_ connected in parallel is:

(8) $$Z=\frac{1}{\frac{1}{Z_{f}}+\frac{\varepsilon }{Z_{0}}}$$

Using VNA determined *S*_11_ and the formula (8) for two media, for example air and acetone, the values of *Z*_0_ and *Z_f_* can be calculated from the following two equations:

(9) $$Z_{\mathrm{air}}=\frac{1}{\frac{1}{Z_{f}}+\frac{\varepsilon_{\mathrm{air}}}{Z_{0}}};\;\;\;Z_{\mathrm{ace}}=\frac{1}{\frac{1}{Z_{f}}+\frac{\varepsilon_{\mathrm{ace}}}{Z_{0}}}$$

where *Z_air_* is the sensor impedance in air, *Z_ace_* is the sensor impedance in acetone, *ε_air_* = 1+*j*0 is the dielectric permittivity of air, *ε_ace_* is the dielectric impedance of acetone determined from Debye model using parameters from Table 1. Finally, the searched dielectric permittivity of the tested medium is calculated from Equation (8).

### 2.4. Model of a Lossy Impedance Transformer—T_T_ Model

**Figure 6.**
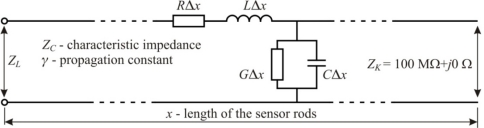
Sensor rods as a transmission line.

For high frequency signals the two-wire sensor should be analyzed as a distributed parameter system, opposite to a lumped parameter system, discussed in previous models. In this case a two-wire sensor is a transmission line composed with four-pole lumped elements (Figure 6) of unit values of resistance *R*, induction *L*, conductivity *G* and capacity *C* for the line unit length Δ*x* [15].

The parameters describing the voltage and current in the transmission line are phase *α* (rad m^−1^), attenuation *β* (dB m^−1^) and propagation *γ* constants as well as characteristic impedance *Z_C_* of a transmission line:

(10) $$\gamma =\alpha +j\beta =\sqrt{\left(R+j\omega L)(G+j\omega C\right)};\;\;\;Z_{C}=\sqrt{\frac{R+j\omega L}{G+j\omega C}}$$

Assuming that soil is a paramagnetic material with relative magnetic permittivity *μ_r_* = 1 and the value of *L* does not depend on soil properties, the unit resistance *R* of the rods becomes zero and the unit value of *C* can be substituted by *C*_0_*ε* and Equation (10) can be substituted by:

(11) $$\gamma =\frac{j\omega }{c}\sqrt{\varepsilon };\;\;\;Z_{C}=\frac{Z_{\mathrm{air}}}{\sqrt{\varepsilon }}$$

where c is light velocity in free space.

The termination of the open line *Z_K_* is modeled as a resistance of the 10 MΩ value. It is transformed to the impedance of the sensor rods beginning as follows [17]:

(12) $$Z_{L}=Z_{C}\frac{Z_{K}+Z_{C}\;\text{tgh}\;\gamma x}{Z_{C}+Z_{K}\;\text{tgh}\;\gamma x}$$

Inserting *γ* and *Z_C_* from Equation (11) to the Equation (12) gives Equation (13) as the impedance *Z_L_* of the sensor rods of the *x* length inserted into the tested medium of known complex dielectric permittivity *ε*. It is much more difficult to determine in the analytical way the transposed function *ε* = *f*(*Z_L_*). However the frequency spectrum of *ε* can be found numerically:

(13) $$Z_{L}=\frac{Z_{K}+\frac{Z_{\mathrm{air}}}{\sqrt{\varepsilon }}\text{tgh}\left(\frac{j\omega x}{c}\sqrt{\varepsilon }\right)}{1+\frac{Z_{K}}{Z_{\mathrm{air}}}\sqrt{\varepsilon }\;\text{tgh}\left(\frac{j\omega x}{c}\sqrt{\varepsilon }\right)}$$

Treating the sensor rods as a lossy impedance transformer enables to find the dielectric permittivity values independent on the length of the sensor rods, but a new problem appears with the ambiguity of the solution. The tangent hyperbolic function is periodic for the imaginary part of the argument. The condition for the multiple solution of the Equation (13) is:

(14) $$\lambda <2\cdot x\;\;\;\text{for}\;\;\;\lambda =\frac{c}{f\sqrt{\varepsilon }}$$

where: c is the velocity of light in free space, *f* – frequency of the applied measurement signal, *λ*— wavelength of the signal in a material of the refractive index $\sqrt{\varepsilon }$, *x* – length of the sensor rods.

**Figure 7.**
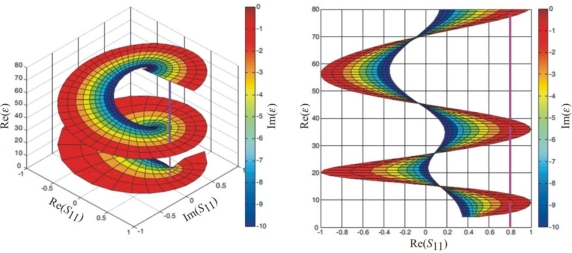
Four-dimension *S*_11_/*ε* chart (complex values) based on the model of lossy impedance transformer (left) and its view in the Re(*ε*)/Re(*S*_11_) plane (right).

If condition (14) is met for the whole range of *Z_L_* values, multiple solutions can be obtained for *ε*. Inserting *Z_L_* as *Z_p_* into Equation (2) it is possible to visualize, with the help of the Matlab R2008b program [16], the relation of the complex function *ε* = *f*(*S*_11_), which is presented in Figure 7. The assumed values of *x* = 10 cm, *f* = 500 MHz. For a line perpendicular to the *S*_11_ plane the values of *ε* are ambiguous. Therefore the numerical solution is enhanced using a tracing procedure that first calculated *ε* at low frequencies, for which Equation (14) assures a single solution. Increasing measurement frequency results in meeting conditions for two or more solutions. To distinguish these solutions the procedure utilizes the former one to apply new limits of the function variability. Such approach enables to interpret the measurements in high frequencies where the wavelength of the measurement signal is shorter than the double length of the sensor rods. The detailed description of applied algorithm and calculation software is presented in [18].

The calibration of the *T_T_* sensor model needs the application of at least three calibration points, for example: (i) impedance of the shorted sensor at the place where the rods are connected with the epoxy resin enclosure (*Z* = 0 + *j*0), (ii) impedance of the sensor in air (*Z* = 1 + *j*0), (iii) impedance of the sensor fully inserted in acetone (or other calibration liquid from the Table 2) calculated from the Debye model. The calibration details are described in [18].

## 3. Materials and Methods

The porous media used for testing the sensors consisted four mineral soils taken from 10–20 cm layer below the ground surface that were characterized by parameters presented in Table 1. The soil data were taken from the data bank of Polish soils [19].

The soils were especially selected to minimize all the factors other than water content and salinity that influence the value of dielectric permittivity, *i.e.*, non swelling or shrinking soils or those with no organic content. There were 10 soil samples with different water content, evenly spaced covering the range from air dry to saturation, for each soil 569, 610 and 622 (Table 1). The water holding capacity was calculated by dividing the weight of the water held in saturated sample by the sample dry weight. Homogeneity of water content in the samples was achieved by sealing the samples and maintaining them at 45 °C during three days.

Because of non-homogeneity of porous materials, especially soil, the liquids of known dielectric parameters (distilled water, acetone, methanol, isopropanol) described by Debye model [20] given by Equation (15) were used to assess the accuracy of the applied FDR probe models (Table 2).

(15) $$\varepsilon =\varepsilon_{\infty }+\frac{\varepsilon_{s}-\varepsilon_{\infty }}{1+j\omega \tau }$$

where *ε*_∞_ and *ε*_s_ are the values of dielectric permittivity above relaxation frequency and in static electric field, respectively, *τ* is the relaxation time of the dielectric medium.

The influence of salinity on the complex dielectric permittivity was tested on 5 series (each with 10 samples) of the black soil 529. The samples were moistened with KCl solutions with variable electrical conductivity values 0, 5, 10, 15 and 20 dSm^−1^. Similarly to the soils 569, 610, 622, the water content in the samples were evenly distributed in steps of 0.1 of the maximum soil water capacity value.

Measurement signal generation as well as detection of the reflected one from the sensor is done by a vector network analyzer (VNA) type ZVCE from Rohde and Schwarz [21] working in the reflection mode in the frequency range 20 kHz – 8 GHz. It measured a complex reflection coefficient *S*_11_ when the sensor rods were fully inserted into the tested material. The ambient temperature in was 20.6 ± 1 °C. According to the instructions, before each series of measurement the VNA was warmed for at least one hour.

**Figure 8.**
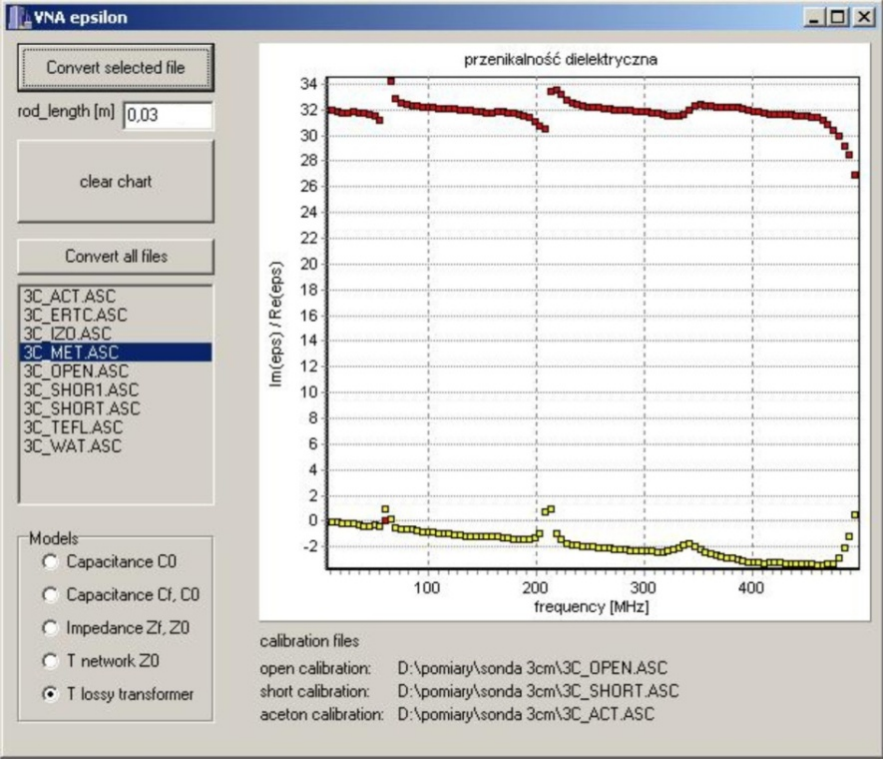
Program window for processing the raw *S*_11_data measured by VNA and applied to the models: *C*_0_ (Figure 3), *C_f_* – *C*_0_ (Figure 2), four-pole *T* (Figure 4), *Z_f_* – *Z*_0_ (Figure 5) and *T_T_* (Figure 6), which are selected by the user.

The raw data *S*_11_ from calibration measurements, as well as final measurements in the tested materials, were processed by a custom software application written in C++ Builder, where the final frequency spectrum of the complex dielectric permittivity was calculated and presented in graphic form. for each of the discussed model. Figure 8 presents the case with selected *T_T_* model. Real and imaginary parts of the complex dielectric permittivity are shown at the upper and the lower part of the diagram, respectively. More details of the application program are available in [18].

Evaluation of the sensor models used to determine the complex dielectric permittivity of tested media were performed by calibration of the sensors in reference liquids (Table 2), measurements by VNA in methanol and verification the calculated real and imaginary parts of the complex dielectric permittivity spectrum with the one calculated with the use of Debye model. The influence of the measurement frequency on the measured refractive index were analyzed for data collected on soil 529 samples using the sensor of 3 cm rods length.

## 3. Results and Discussion

### 3.1. Model Selection

**Figure 9.**
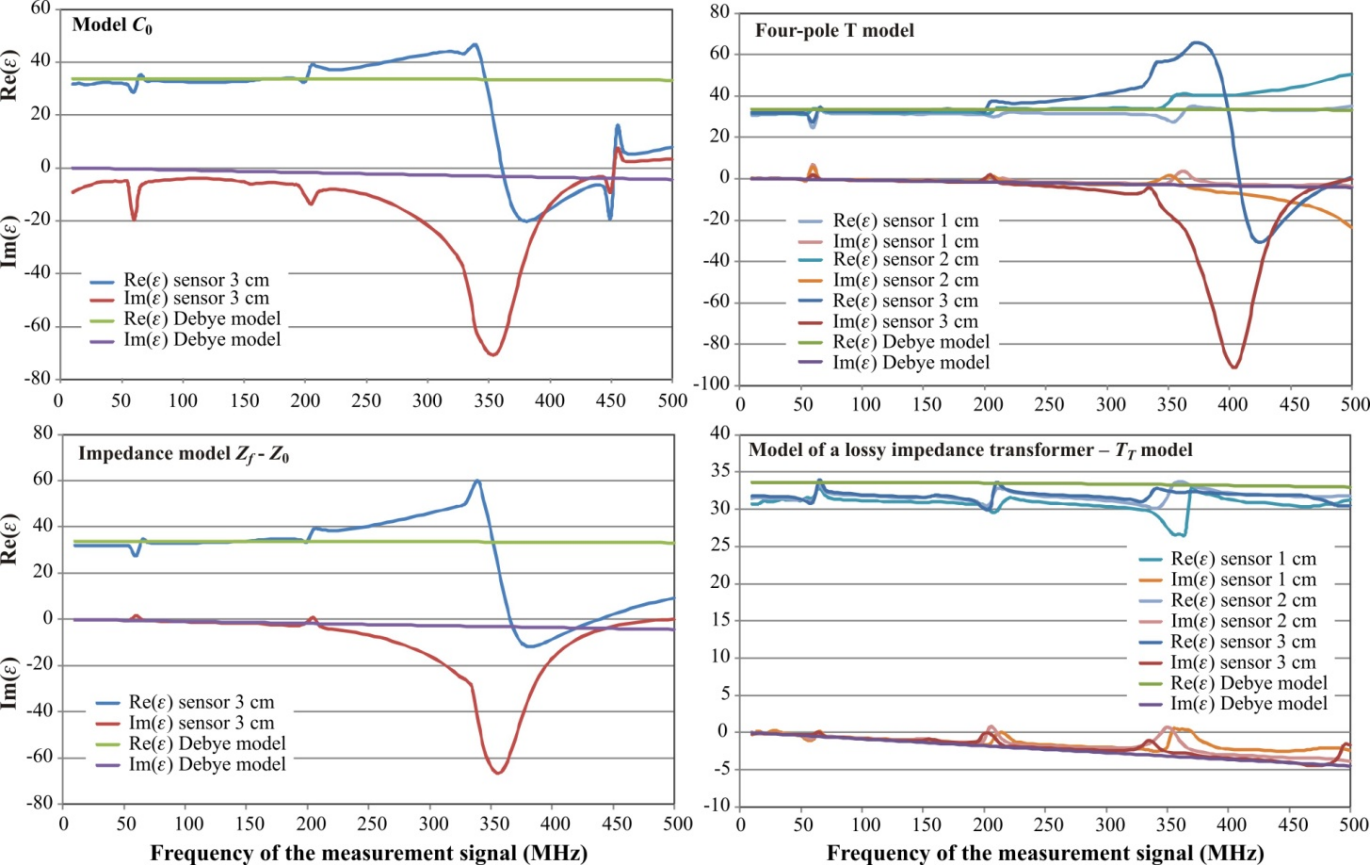
Performance comparison of the analyzed FDR sensor models for methanol as the verification medium.

The comparison of the complex dielectric permittivity values calculated on the base of calibration measurements in reference liquids for various models described in earlier sections is presented in Figure 9.

**Figure 10.**
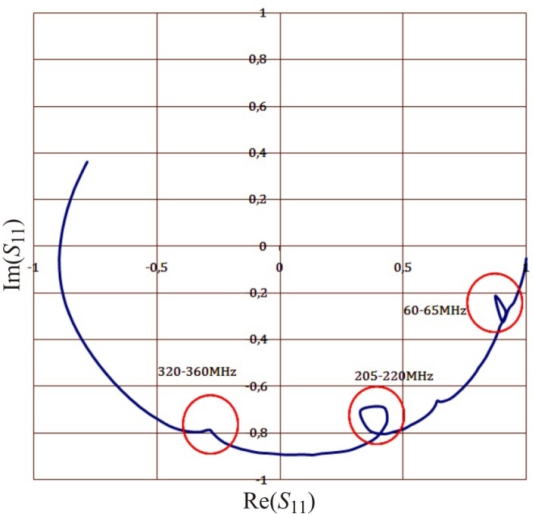
Resonance effects (in circles) on the *S*_11_ parameter measured by VNA.

The artifacts on each graph (Figure 9), as well as in the respective graph of the complex reflection coefficient *S*_11_ measured by VNA observed for the same frequencies (Figure 10), are discussed in [22] for open-ended coax sensors. They are caused by resonance effects in an open transmission line. This happens when the length of a cable with a sensor equals to the odd multiple wavelength of the measurement signal. This was confirmed in separate tests not presented in the current study. These effects resulted in limiting the frequency range of the measurement signal to 10–500 MHz.

It is evident from Figure 9 that the model *C*_0_ can be successfully used only for the correct determination of the real value of the complex dielectric permittivity Re(*ε*) for the frequencies up to 200 MHz. The imaginary part Im(*ε*) has the measurement error above 10% for all applied frequencies. Similar results were found for the model *C_f_* – *C*_0_ (Figure 2), which was not presented in Figure 9. This can be explained by a minimal influence of the *C_f_* capacitance in the two-wire sensor.

Better results were found for the impedance model *Z_f_* – *Z*_0_, especially for the imaginary part Im(*ε*). However the useful frequency did not increase above 200 MHz. The graphs in Figure 9 concerning the models *C*_0_ and *Z_f_* – *Z*_0_ only refer to the sensor length equal to 3 cm, because similar results were observed for other sensor length 1 cm and 2 cm.

The four-pole T model performs better for both components of the dielectric permittivity, but for a 3 cm length probe the upper measurement frequency limit is still around 200 MHz and 350 MHz for the 2 cm length sensor. This model correctly follows the values of Debye model calculated for methanol as the reference liquid for the sensor of 1 cm rod length.

The best performance was observed for the model of lossy impedance transformer (*T_T_* model). The real part of the dielectric permittivity is not dependent on the sensor length for the whole frequency range of the measurement signal. Only a slight decrease of results are observed, especially for the real part. This effect can be explained by the probable increase of dielectric permittivity of acetone used for the sensor calibration. The sensor length affects the performance of the imaginary part of the model and the length is shorter, the measurement error of Im(*ε*) is bigger.

The combination of calibration points of the *T_T_* model also influenced the measurement error of *ε* for both components of the dielectric permittivity. The best results were obtained for the combination of water, air and acetone, where for √Re(*ε*) < 2 the measurement error of the real part of dielectric permittivity is less than 4% and it is below 2.2% for √Re(*ε*) ≥ 2.

**Figure 11.**
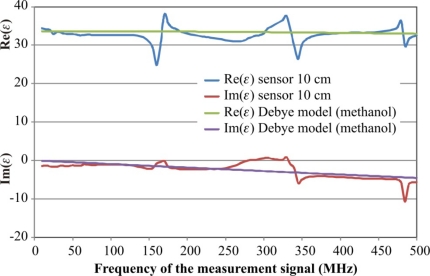
Performance of *T_T_* model for the FDR sensor with 10 cm length of rods.

The *T_T_* model was applied to the sensor of 10 cm length of parallel metal rods and its performance is presented in Figure 11. In the frequency range 125–375 MHz the real part of dielectric permittivity of methanol has a big maximal measurement error reaching 20% of the measured value, but its average value is very close to the model value. The imaginary part of *ε* behaves similarly. The erroneous effect can be explained with the resonance of the open line described earlier in Figure 10 and they increase with the increase of the sensor rods’ length. It seems that the *T_T_* model could be applied for 10 cm length sensors which have bigger measurement volume as compared with 1–3 cm long ones. However before that it is necessary to eliminate the resonance effect and develop a new calibration methodology of these sensors.

### 3.2. Influence of Measurement Frequency on the Measured Refractive Index

Analysis of the measurement frequency influence on the measurement result was done by the comparison of the mean values of the refractive index for six selected frequency bands. The refractive index defined as the square root of the real part of the dielectric permittivity was calculated from the *T_T_* model of a lossy impedance transformer. The selected frequency bands were: 10–30, 35–50, 80–120, 125–190, 245–300, 390–485 MHz. These bands were not burdened with errors caused by the open transmission line resonance described in the previous section.

**Figure 12.**
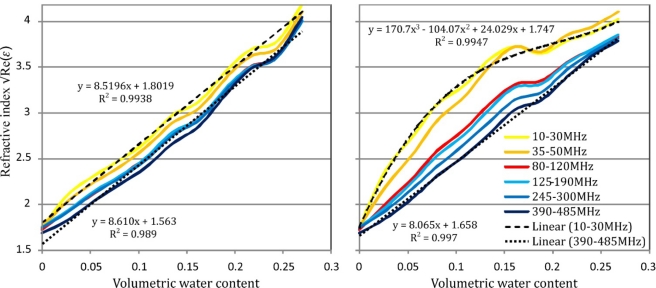
The influence of the measured frequency range on the value of the soil refractive index for the samples of soil 529 wetted with distilled water (left-hand graph) and 20 dSm^−1^ KCL solution (right-hand graph).

Figure **12** presents the relation between the soil refractive index √Re(*ε*) and volumetric water content for the soil 529 for various frequency ranges.

For soil samples wetted with distilled water the refractive index increases with the frequency decrease for the whole range of soil moisture and the trend lines representing various frequency ranges are nearly in parallel (Figure **12**—left). Trend lines for all frequency ranges have the determination coefficient R^2^ not less than 0.989. For figure clarity, only extreme trend lines are presented.

For soil samples wetted with KCl solution of the electrical conductivity equal to 20 dSm^−1^, the lower the measurement frequency, the bigger was the refractive index of soil samples with the same thermogravimetric water content. In the frequency range 390–485 MHz the relation can be described by a linear relation with determination coefficient equal to 0.997. For the frequency range 10–30 MHz this relation is extremely far from linear and must be fitted with the polynomial of the 3^rd^ degree to achieve R^2^ = 0.994. This means that for the tested salted sandy soil low frequency dielectric measurements of soil moisture require soil specific calibrations. However, it should be noted that the shape of this calibration still depends on the salinity level.

### 3.3. FDR Soil Moisture Calibration

The analysis included data for the FDR sensor with 3 cm rod length and 390–485 MHz frequency range of the measurement signal, for which the real part of dielectric permittivity does not show any frequency dependence. Data for 20 measurement points of Re(*ε*) from this range were averaged for each soil sample to calculate the square root as the refractive index. The standard deviation for all measurements did not exceed 0.16 of the absolute value. The correlation of final data with the corresponding thermogravimetric data is presented in

The fitted linear functions are presented for comparison with literature data. The trend line fitted to the all data points is: $\sqrt{\text{Re}(\varepsilon )}=a_{0}\theta +a_{1}$, where *a*_0_ = 8.86, *a*_1_ = 1.48.

The values of the *a*_0_ and *a*_1_ coefficients determined for the Malicki model from the TDR calibration measurements are very close to the ones from the presented FDR calibration. This is especially true for density corrected calibration. This means that in the measurement frequency range of 390–480 MHz the soil moisture values determined for presented FDR sensor and the applied sensor model are comparable with the TDR ones.

## 4. Conclusions

The presented two-wire FDR sensors and the measurement methodology of the frequency spectrum of the complex dielectric permittivity enable simultaneous and selective determination of soil moisture for various frequency ranges of the measurement signal. The developed lossy impedance transformer—*T_T_* model of the FDR two-wire sensor can be used to measure the frequency spectrum of soil complex dielectric permittivity in the range 10–500 MHz, that up till now is not used in commercial FDR meters of soil moisture and salinity. It was found that for the FDR measured soil samples the real values of the complex dielectric permittivity determined for low frequencies (10–50 MHz) are higher in relation to the ones determined for high frequencies (300–500 MHz), especially for saline soils. The soil moisture values determined for the chosen mineral soil samples by the applied FDR method and sensors are comparable to the ones determined by the TDR method.

## Figures and Tables

**Figure 13.**
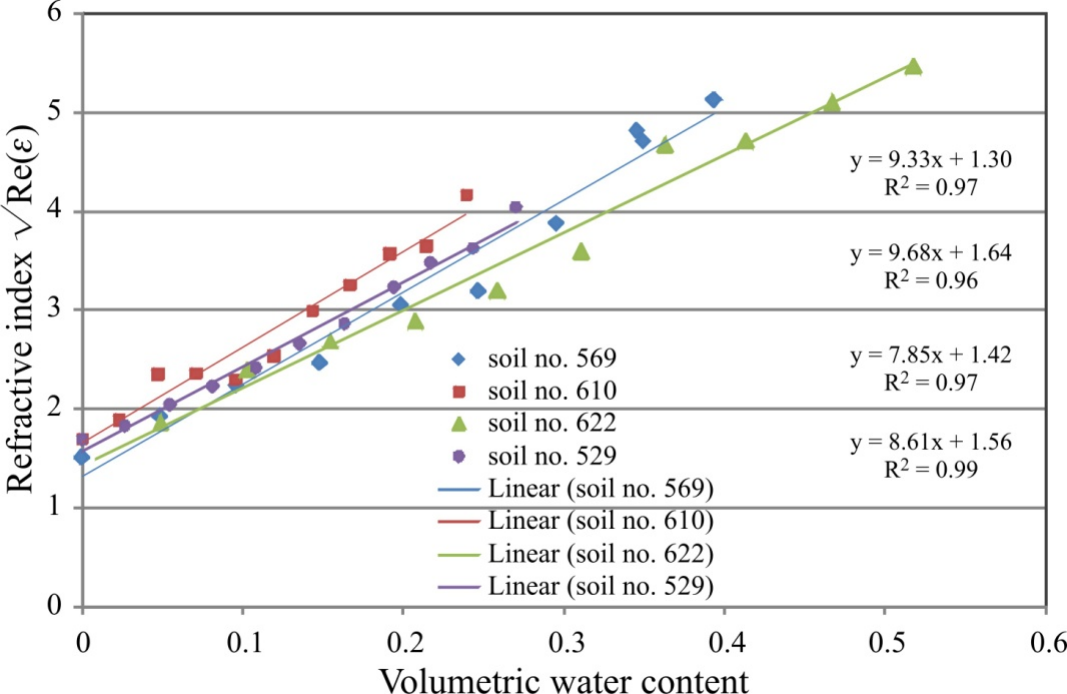
FDR soil moisture calibration for the measurement frequency range 390–480 MHz.

**Table 1.** Localization and selected physical parameters of tested soils.

No.	Soil type	Soil localization	Dry bulk density (gcm^−3^)	Soil texture by FAO (%)			Specific surface (m^2^g^−1^)	Water holding capacity (kg kg^−1^)
				Sand	Silt	Clay		
569	Brown	Majdan Skierbieszowski	1.33	71	25	4	21	0.39
610	Brown	Kol. Olempin	1.59	94	5	1	9	0.13
622	Chernozem	Terebin	1.4	60	34	6	37	0.36
529	Black soil	Annopol	1.76	100	0	0	8	0.16

**Table 2.** Parameters of Debye model of the applied reference liquids [20].

Name	Temperature (°C)	*ε*_s_	*ε*_∞_	*τ* (ps)
Distilled water	20	80.4	5.2	9.45
Acetone	20	21.2	1.9	3.34
Methanol	20	33.64	5.7	53
Isopropanol	20	29	3.2	292

**Table 3.** Comparison of FDR calibrations of the analysed sensors with Malicki TDR calibrations [23].

	$\sqrt{\text{Re}(\varepsilon )}=a_{0}\theta +a_{1}$		
	FDR measurements (Figure **13**)	Malicki model	Malicki model (correction on soil density)
*a*_0_	8.86	7.16	8.96
*a*_1_	1.48	1.44	1.44
